# Utilization of LED Grow Lights for Optical Wireless Communication-Based RF-Free Smart-Farming System

**DOI:** 10.3390/s21206833

**Published:** 2021-10-14

**Authors:** Sana Javed, Louey Issaoui, Seonghyeon Cho, Hyunchae Chun

**Affiliations:** Department of Information and Telecommunication Engineering, Incheon National University, Incheon 22012, Korea; engr.s@inu.ac.kr (S.J.); loueyissaoui@inu.ac.kr (L.I.); seonghyeon.cho@inu.ac.kr (S.C.)

**Keywords:** Li-Fi, smart farm, optical camera communication, optical wireless communications, visible light communications, OWC, VLC

## Abstract

Indoor smart-farming based on artificial grow lights has gained attention in the past few years. In modern agricultural technology, the growth status is generally monitored and controlled by radio-frequency communication networks. However, it is reported that the radio frequency (RF) could negatively impact the growth rate and the health condition of the vegetables. This work proposes an energy-efficient solution replacing or augmenting the current RF system by utilizing light-emitting diodes (LEDs) as the grow lights and adopting visible light communications and optical camera communication for the smart-farming systems. In particular, in the proposed system, communication data is modulated via a 24% additional green grow LED light that is also known to be beneficial for the growth of the vegetables. Optical cameras capture the modulated green light reflected from the vegetables for the uplink connection. A combination of white ceiling LEDs and photodetectors provides the downlink, enabling an RF-free communication network as a whole. In the proposed architecture, the smart-farming units are modularized, leading to flexible mobility. Following theoretical analysis and simulations, a proof-of-concept demonstration presents the feasibility of the proposed architecture by successfully demonstrating the maximum data rates of 840 b/s (uplink) and 20 Mb/s (downlink).

## 1. Introduction

With ever-increasing environmental issues, the importance of securing healthy food has been widely acknowledged as one of the most crucial problems in the near future. Therefore, new technologies and techniques to get the maximum yield has been actively investigated. In light of this, new agriculture techniques and types have been introduced. One of them is called Agriculture 4.0, in which the vegetables growing under artificial lights revolutionized the way of traditional agriculture [1]. For instance, light intensity, spectrum controlling, and photoperiod are directly related to vegetable growth, productivity, and size. Therefore, a number of actuators and sensors are installed across the farming area to meet the required necessities and environmental conditions. Such complex management can be simplified with advanced agriculture techniques, leading to a low cost and high productivity, independent of the season.

In reference [2,3], the authors proposed a Wi-Fi-based wireless network for data acquisition, intelligent management, and control of the farming area. In reference [4], the LoRaWAN network was deployed not only for farm supervision but also for real-time monitoring of local weather in remote areas. Moreover, in reference [5], a ZigBee-based sensor network was proposed for checking the conditions for agriculture. In the architecture, the humidity sensors, temperature sensors, lights, and different actuators were connected to wireless modules for uploading the data on the cloud [6]. Bluetooth was also deployed in agricultural projects. In reference [7], plowing, cup-cutting, pesticide-spraying, and seeding applications were done by a robot via Bluetooth. Some other agricultural management systems currently are in the market, where sensors transmit data through a 920 MHz wireless channel for the uplink, and 3G/LTE channel is adopted for the downlink [8]. These up-to-date smart management systems in the agriculture field rely on the radio-frequency (RF) spectrum as a medium for data transmission.

However, some experiments showed the impact of RF radiation from Wi-Fi routers on the germination of the seeds, and the growth of the vegetables could be detrimental [9,10]. It was reported that seeds exposed for a longer time to the RF radiation took a much longer time to germinate or failed to grow. In addition, the vegetables exposed to the radiation showed lesser and brighter leaves and were shorter in size than those not exposed to the radiation. The work showed that conventional Wi-Fi routers could affect the life cycle of vegetables, causing a decrease in their fruit quality and the photosynthesis process.

With these possible side-effects from RF-based indoor smart-farming systems, visible light communication (VLC) has been gaining attention as a promising candidate for replacing or augmenting the current RF-based systems [11]. VLC typically uses light-emitting diodes (LEDs) as light sources and data transmitters, providing the simultaneous functions of illumination and communication [12]. Reference [13] presented that LEDs can provide the required energy for photosynthesis which is the primary process for the growth of vegetables. The work also reported that the LEDs showed maximum energy efficiency and reduced cost compared to other artificial grow lights. In addition, reference [14] investigated the energy consumption of VLC compared to RF technologies and showed VLC’s superiority in energy efficiency. Another study proved that VLC is a safer, faster, greener, and cheaper technology [15]. Therefore, VLC can be an attractive solution by generating lights for growing vegetables and constructing a communication network for smart-farming.

In this work, we propose a new smart-farming architecture guaranteeing an RF-free growing environment for plants to increment crop production and ensure the best quality of products. The proposed system is entirely RF-free, energy-efficient, and flexible. For making the system energy-efficient, a well-collimated RGB downlight to the vegetables is utilized. This downlight provides a well-defined photon flux for growing leafy vegetables and also allows the reflected light to be reused for communication. In this work, leafy vegetables such as lettuce are chosen to investigate since it is among the most studied species in indoor smart-farming using LEDs [16]. For growing lettuce, although the conventional red-blue LED combination is still effective, additional 24% green LED light can enhance the growth of the vegetable [17]. Vegetables have their own reflectance towards each color. Considering that the green light has the highest reflectance, here we propose to use the modularized concentrated green reflected light for the uplink communication. For a complete RF-free environment, a downlink constructed from the combination of white LEDs (ceiling lights) for workers in the farm and a photodiode installed in each smart-farming unit is investigated. The modularized and relocatable smart-farming units allow various types of vegetables to grow in the same unit farm.

In Section 2, a description of the proposed smart-farming architecture and fundamentals of the photosynthetic photon flux density and its connection with communication parameters are presented. Section 3 describes the findings from a series of simulations, followed by proof-of-concept demonstrations showing the feasibility of the proposed architecture. Finally, discussions and conclusions are drawn in Section 4.

## 2. Proposed Architecture and Fundamentals of PPFD for Communication

### 2.1. Proposed Energy-Efficient Smart-Farming Architecture

**Figure 1:**
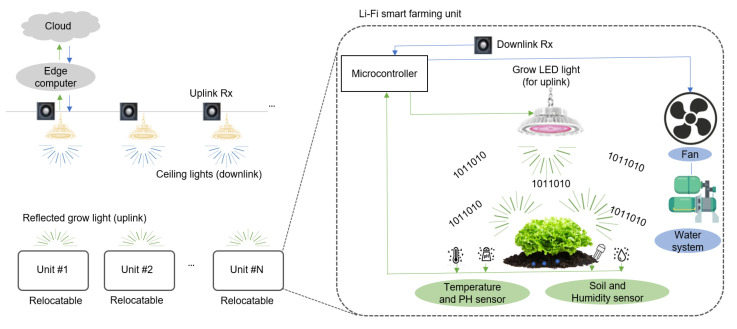
Proposed energy-efficient smart-farming architecture.

Figure 1 shows the proposed energy-efficient smart-farming architecture. The architecture consists of several relocatable smart-farming units, white LEDs for ceiling lights, uplink receivers, the edge computer, and the cloud.

For making a complete RF-free environment, uplink and downlink communication utilize VLC technology. For the uplink, modulated green LED lights from the smart-farming unit are detected by an uplink receiver deployed above the vegetables near the ceiling lights. As the main computing unit, the edge computer monitors and controls the smart-farming units. According to the processed data, appropriate commands are made and sent back to each smart-farming unit through the downlink. For the downlink, the ceiling lights installed for a convenient working environment are reused as a transmitter. A downlink receiver placed near the grow LED lights receives the signal. To store the data for long-term use, the edge computer uploads it to the cloud.

In this indoor smart-farming application, each smart-farming unit is composed of grow LED lights, a microcontroller, a receiver for downlink, and several sensors and actuators. A well-collimated RGB LED downlight is considered as the grow light for the vegetables. A key feature of the proposed smart-farming unit is that the green modulated light reflected from the leafy vegetables after photosynthesis is reused for uplink communication. Sensors monitor parameters such as PH, humidity, temperature, soil, and intensity. Actuators such as water pumps, fans, lamp drivers perform the action on the commands. All these sensors and actuators are connected to the microcontroller. In the case of uplink communication, the microcontroller sends the data recorded by each sensor to the edge computer through the uplink constructed by the modulated green LED light reflected from the leafy vegetables. This information is received by the uplink receiver placed near the ceiling lights. The edge computer processes the received data and generates the appropriate commands. In the case of downlink, such commands are sent back to the microcontroller using ceiling lights. The downlink receiver near the grow lights receives the signal. Based on these received commands, the microcontroller activates the required actuator devices. 

Since wired connections are limited in location and mobility, one of the main advantages of using wireless communication is achieving such locational flexibility for each smart-farming unit. Although the wired connections throughout the farming area can allow the RF-free environment, this can cause complex cable installation, difficulty in maintenances, and less flexible mobility of the individual farming unit.

### 2.2. PPFD to Illuminance Conversion

The spectra of LED lights and the absorptance of Chlorophyll A and B that dominate the photosynthesis process are crucial factors. In photosynthesis, the blue and the red spectra are primarily used for growing vegetables. However, an experimental investigation suggested that the addition of 24% green LED light can enhance the growth of the vegetable [17]. This addition of green light has a better penetration in the vegetable canopy and increases vegetable growth by potentially improving the photosynthesis process. Moreover, it solved the problems of complex visual assessment stemming from the red-blue combination creating the purplish-gray light. Thus, the additional green light is also taken into account as a grow light and communication source in this work.

For the growth of the indoor vegetable, a certain energy level required for the process of photosynthesis has to be met. The photosynthetic photon flux density (PPFD) is the most frequently used unit to analyze the required photosynthesis level. The unit of PPFD is μmol·m^−2^·s^−1^, and it implies the amount of photosynthetically active radiation (PAR) arriving at the vegetable. It can also be thought of as the amount of photons in μmol falling on a unit area for a second. Illuminance (with the unit of lux) is a more frequently used term in visible light communication. However, PPFD is not a straightforward definition to be converted into illuminance. For instance, an illuminance of 54 lux is equal to is 1 μmol·m^−2^·s^−1^ when sunlight is considered [18]. However, for artificial lights with various spectral distributions, the relationships have to be carefully derived. 

The calculation of the available photosynthetic energy to vegetables is possible if the spectral power distribution (SPD) of a light source is known across the relevant visible wavelengths (400–700 nm). It is defined that 683 lumens of luminosity flux are equals to 1 watt of radiant power at 555 nm. Using these values and the CIE 1931 luminous efficiency function, the luminous flux (in lumen) can be obtained [19]. Then, the spectral radiant flux *Φ*(*λ*) in W/nm per lumen can be calculated as:

(1) $$\frac{\Phi \left(\lambda \right)}{\mathrm{lumen}}=\frac{P_{rel}\;\left(\lambda \right)}{683\cdot \sum_{400nm}^{700nm}\;V\left(\lambda \right)\cdot P_{rel}\left(\lambda \right)\cdot \Delta \lambda }$$

where *V*(*λ*) is the luminous efficiency function at *λ* wavelength and *Prel* (*λ*) is the relative spectral power distribution. The denominator indicates the overall luminous flux. For instance, a light source with a center wavelength closer to 555 nm leads to a higher luminous flux and vice versa. The relative spectral radiant flux is used for PPF-lumen conversion. Then the photosynthetic photon flux per wavelength in μmol·s^−1^ nm^−1^ is calculated as [20]:

(2) $$\frac{\mathrm{PPF}}{\mathrm{nm}}=\frac{10^{-9}\;\cdot \;\lambda \cdot \Phi \left(\lambda \right)}{N_{a}\cdot h\cdot c}$$

where *λ* is the wavelength (in *m*), *N*_a_ and *h* are the Avogadro’s constant (6.022 × 10^23^) and Planck’s constant (6.626 × 10^−34^), respectively, and *c* means the speed of light. Therefore, for a given light source, the photosynthetic photon flux (PPF) per lumen can be obtained when its spectral radiant flux (*Φ*(*λ*)) is given. Then, the PPFD of the source can be converted to the illuminance value (lux = lumen/m^2^). Table 1 shows the calculated Lux-PPFD conversion factors for the commercial LED light sources used in this work.

### 2.3. Design Parameters for Illumination and Communications 

In designing and selecting the LEDs to achieve the required illuminance and the communication performance, the luminous efficacy of the light source should be known. In VLC, the illuminance is a typical constraint limiting the optical power available for communication. Therefore, it is important to choose the optimum luminous efficacy. The luminous efficacy (*η*) is defined as [21]:

(3) $$\eta =\frac{\Phi }{P}\;\;\left[\frac{\mathrm{lm}}{w}\right]$$

The luminous efficacy indicated how much luminous flux (*Φ*) is included in a watt of radiant power flux (P) and has the unit of lm/W. Table 2 shows the calculated luminous efficacy for the commercial REB LEDs used in this work. The Lambertian order (*m*) of the light source is also important, which shows the degree of divergence, which is defined as [22]:

(4) $$m_{\;}=\frac{-\ln\left(2\right)}{\ln(\cos\Phi_{1/2})}$$

where *Φ*_½_ is the semi-angle for the half intensity. For the perfect Lambertian emission, the order *m* = 1 and the semi angle becomes *Φ*_½_ = 60°. The calculation of luminous intensity (R(φ)) can be found by [23,24]:

(5) $$R\left(\varphi \right)=\Phi \cdot \left[\frac{\left(m+1\right)}{2\pi }\right]\cdot \cos^{m}\left(\varphi \right)\;\;\left[\frac{\mathrm{lm}}{\mathrm{sr}}\right]$$

where *φ* represents the radiation angle. The unit of the angular intensity is lm/sr, which is equal to a candela, 1 cd. The conversion of luminous intensity to luminous flux is calculated by [25]:

(6) $$E=R\left(\varphi \right)\cdot \frac{\cos\theta }{r^{2}}\;\left[\mathrm{lux}\right]$$

where *E* is the luminous intensity defined as the amount of lumen on a unit area. Here, *θ* denotes the angle between the normal direction of the illumination plane and the direction of light propagation. *r* means the distance between the light source and the illumination plane. The light intensity is reduced as the distance increases. Most of the vegetables are placed at a distance of 6 to 12 inches (15~30 cm) from the light source [26].

The PPFD required for the growth of the leafy vegetables is mentioned to be 200 μmol·m^−2^·s^−1^ to 300 μmol·m^−2^·s^−1^. This range generally depends on the target biomass and productivity [27,28]. Reference [29] reported that the progressive increase in the biomass of the lettuce is observed up to 250 μmol·m^−2^·s^−1^_,_ but no further improvement afterward. Therefore, the PPFD level of 250 μmol·m^−2^·s^−1^ is chosen for the following simulations and experiments. Table 3 summarizes the required PPFD for the lettuce. The optimum ratio of PPFD for red, green, blue LEDs is 61%, 24%, and 15%, respectively [17]. The corresponding illuminance and the intensity are derived by the conversion factors shown in Table 1 and Table 2.

## 3. Results

### 3.1. Simulation Results

**Figure 2:**
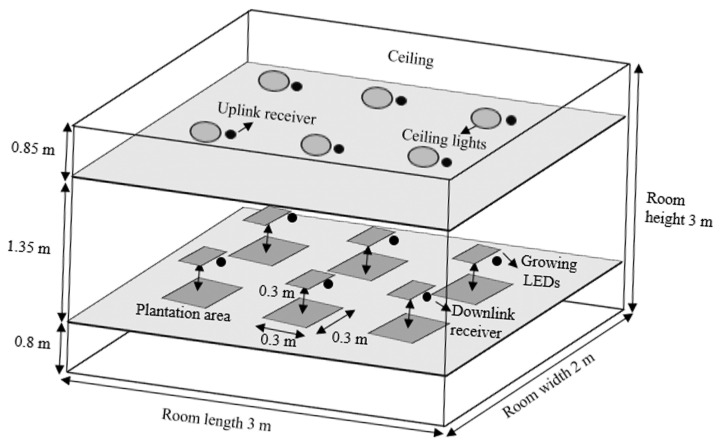
Geometric representation of the simulation setup.

To investigate the available optical power and channel loss for communication in the proposed smart-farming architecture, an analysis with a series of simulations is executed. In this investigation, a farming room of 3 m × 2 m × 3 m is assumed. Figure 2 demonstrates the geometrical representation of the room. 

For the downlink communication, the ceiling lights inside the farming room are utilized. The ceiling light units are assumed to be at the height of 2.15 m from the ground and consist of six units. The leafy vegetables are located at the height of 0.8 m (typical desk level) from the ground. Six grow light units are located at the height of 1.1 m (0.3 m above the vegetable.) The uplink and downlink receivers are assumed to be placed at the same height as the ceiling light units and the grow light units, respectively. Other detailed geometrical and optical parameters are summarized in Table 4.

**Figure 3:**
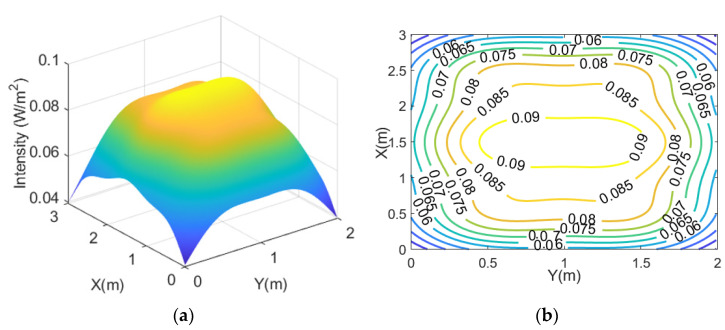
(**a**) Optical channel loss per unit area for the downlink; (**b**) its contour plot.

Firstly, the optical channel loss of downlink from the ceiling lights to the plane of downlink receivers is simulated. For this relative investigation, 1 W of radiant flux per ceiling light unit is assumed. The simulation results are shown in Figure 3a,b and indicate that the minimum, average, and maximum intensity available are 0.04 W/m^2^, 0.08 W/m^2^, 0.09 W/m^2^, respectively. This means there is an optical channel loss of ~11 dB per unit area on average. When a photodetector with an active area of 1 cm^2^ is used, an optical channel loss of ~31 dB on average is obtained. The standard deviation across the receiver plane is just 0.01 W/m^2^ (~13% of the average), enabling a reasonably even downlink performance at any receiver location.

**Figure 4:**
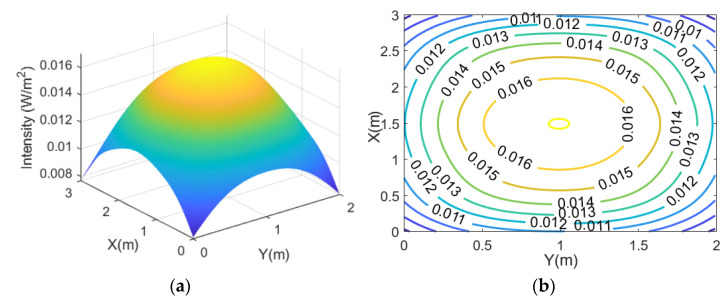
(**a**) Optical channel loss per unit area for uplink without blockage from grow light fixture; (**b**) its contour plot.

Next, the uplink performance is evaluated. Vegetables have their own reflectance in each growing stage. In the maturity stage, the mean value of reflectance for red, green, and blue are 0.121, 0.22, and 0.067, respectively [30]. Due to its relatively high reflectance, the green light can be a good candidate for uplink communication. In this simulation, the modulated green LED light is reflected by the leafy vegetables, and it is scattered upwards. It is assumed that the vegetables act as Lambertian sources. The modulated and reflected light is then detected by an uplink receiver at 1.35 m above. Figure 4a,b show the intensity distribution. The simulation results show that the minimum, average, and maximum intensity achievable are 0.0077 W/m^2^, 0.0137 W/m^2^, 0.017 W/m^2^, respectively. It means there is an optical channel loss of ~18.6 dB per unit area on average. Also, the standard deviation across the uplink receiver plane is 0.0016 W/m^2^ (~12% of the average). Although the evenness is slightly worse than the case of downlink, the standard deviation is still considered reasonably small, allowing a reasonably similar uplink performance at any uplink receiver location except for the corners.

**Figure 5:**
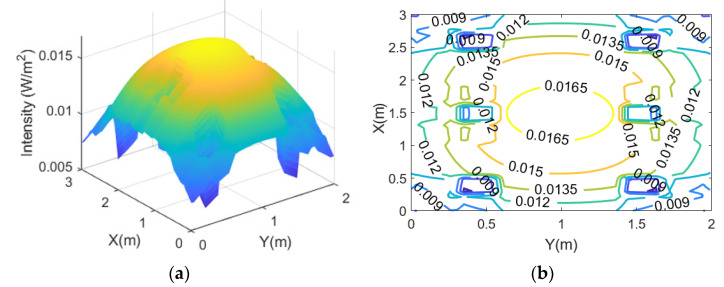
(**a**) Optical channel loss per unit area for uplink with blockage from grow light fixture; (**b**) its contour plot.

Unlike the downlink, however, the uplink channel can be blocked by the grow light units as they are located right above the vegetables. The impact of this line-of-sight (LOS) blockage is simulated by placing ray-stopping objects with a size of 0.2 m × 0.2 m at 0.3 m above the vegetables. The size and height are chosen from the practical dimensions of RGB LED units and recommended distances [26]. In the simulation, each ray passing through the locations of ray-stopping objects is terminated. Figure 5a,b present the estimated optical channel loss per unit area in this case. The results are similar to the case without the blockage except that there are clear shadows under the fixed grow LED units. The minimum (at the shadow areas), average and maximum values are 0.0058 W/m^2^, 0.0129 W/m^2,^ and 0.017 W/m^2^, respectively. The average optical channel loss is ~19 dB. The standard deviation across the receiver plane is still 0.0017 W/m^2^ (~13% of the average), which is slightly worse than the case without the blockage. The shadow areas still have ~34% of the maximum value due to the contributions of the uplink lights from the other smart-farming units. When only a single, smart farming unit is considered in the room, the shadow region is completely blocked. Therefore, choosing the right location for the uplink receiver becomes essential.

### 3.2. Proof-of-Concept Demonstration

**Figure 6:**
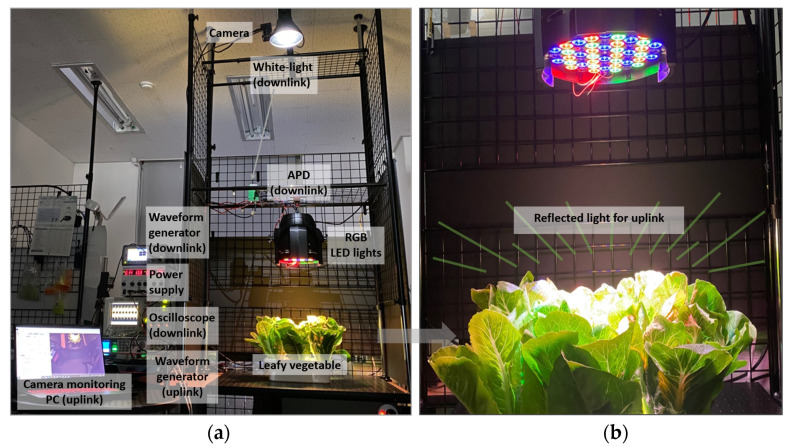
(**a**) Experimental setup; (**b**) reflected light from the vegetable for uplink communications.

To test the feasibility of the proposed smart-farming system, a proof-of-concept demonstration with a single smart-farming unit is demonstrated. Figure 6a shows the experimental setup for the uplink and downlink communications. For downlink communication, a waveform generator (Keysight, 33500B, Santa Rosa, CA, USA) creates a pulse amplitude modulation (PAM) with two levels (2-PAM), also known as on-off-keying (OOK). The signal is combined with DC bias using a bias-T (Mini-circuit, JEBT-4R2GW, Brooklyn, NY, USA) and applied to a commercial white-light LED (downlink light). An APD near the RGB LED lights (KHL, LED54 Gupar Light 162W, Seoul, Korea) receives the signal and is analyzed on the oscilloscope (Keysight, DSO6104, Santa Rosa, CA, USA). For the uplink communication, the waveform generator (Keysight, 81150A, Santa Rosa, CA, USA) generates the PAM signal, and it is applied to the green LEDs. The blue and red LEDs are connected to a DC power supply (Smart, RDP-303AU, Carlsbad, CA, USA) for generating the grow lights. An optical camera (Thorlabs, Zelux 1.6 MP CMOS, Newton, NJ, USA) placed near the white-light is used to detect the signal and is analyzed on the camera monitoring PC. Figure 6b shows reflected RGB lights from the vegetable surface for the uplink communication.

**Figure 7:**
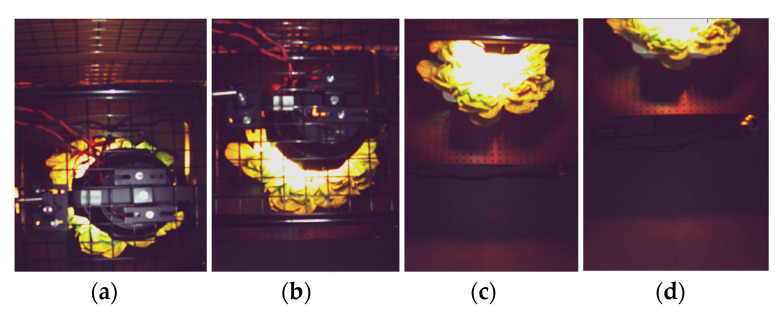
Camera image at the distance of (**a**) 0 cm; (**b**) 25 cm; (**c**) 50 cm; (**d**) 85 cm from the vegetable.

Figure 7 presents the optical camera image at the distance of 0 cm, 25 cm,50 cm, and 85 cm away from the center. At 0 cm (center-aligned), most leaves are covered by the grow light unit, where less reliable communication performance is expected. At 25 cm and 85 cm, almost half of the vegetables are exposed due to the LOS blockage and being outside the camera’s field of view (FOV), respectively. The camera can capture the majority of the reflected light at 50 cm.

#### 3.2.1. Downlink Communication Performance

**Figure 8:**
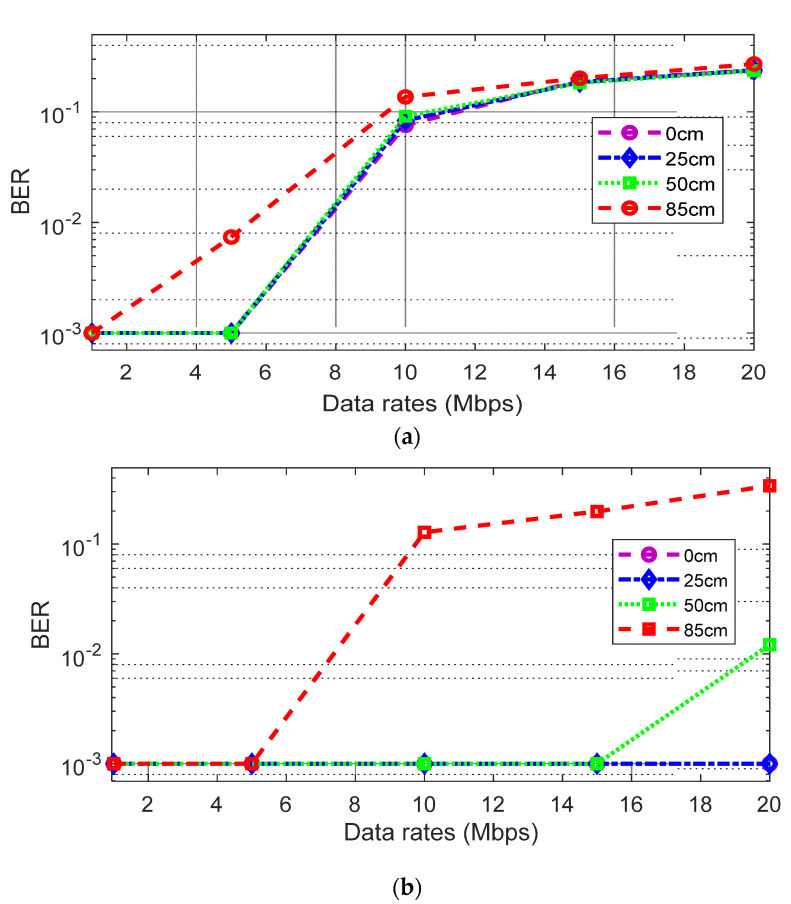
(**a**) BER using 2-PAM (without equalizer) at distance of 0 cm, 25 cm, 50 cm and 85 cm; (**b**) BER using 2-PAM (with equalizer) at distance of 0 cm, 25 cm, 50 cm and 85 cm.

For the downlink communication, Figure 8a shows the bit-error-rate (BER) performance using the 2-PAM scheme. BERs with various data rates are measured at four different distances. The results show that BER gets worse by increasing the distance. This is because the downlink light intensity is decreased by the distance. The result at 85 cm is the worst, achieving only a Mb/s link with less than 10^−3^ BER (the minimum measurable error floor in this test). The data rate increased to 5 Mb/s at distances of 0 cm, 25 cm, and 50 cm, showing a similar tendency. It is mainly because the impact of inter-symbol-interference (ISI) due to the limited bandwidth of the white-LED light is the dominant factor degrading the BER performances.

**Figure 9:**
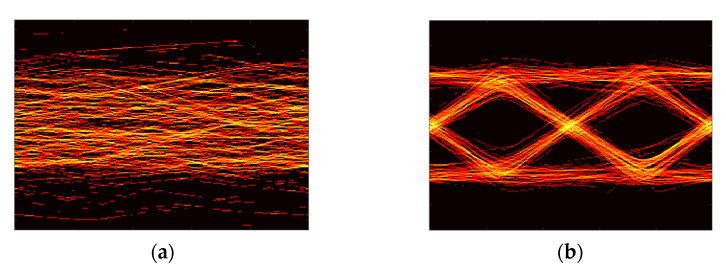
(**a**) Eye-diagram of the received 2-PAM (OOK) signal of 20 Mb/s for the downlink communication at the distance of 25 cm; (**b**) that with DFE.

Figure 8b shows the BER performance with a decision feedback equalizer (DFE) to compensate for the limited bandwidth. Overall, the BER performances are improved, leading to less than 10^−3^ BER at 5 Mb/s, 15 Mb/s, and 20 Mb/s for the case of less than 85 cm, 50 cm, and 25 cm, respectively. Figure 9a,b show the eye diagrams before and after applying the DFE at 25 cm, with the data rate of 20 Mb/s. It can be seen that there is no clear eye-opening because of the severe ISI in the received signal. However, after applying the DFE, a clear eye-opening is observed, showing a BER less than 10^−3^.

#### 3.2.2. Uplink Communication Performance

For uplink communication, firstly, an optical camera monitors multiple smart-farming units with a full region-of-interest (full-RoI) using full pixels. A faster communication link can be constructed by tracking a specific unit by limiting the FoV but increasing the frame rate (tracked -RoI) using partial pixels. The frame rate from the camera is 34.8 fps and 849.6 fps for the full-RoI (1440 × 1080 pixels) case and tracked-RoI (80 × 4 pixels) case, respectively.

**Figure 10:**
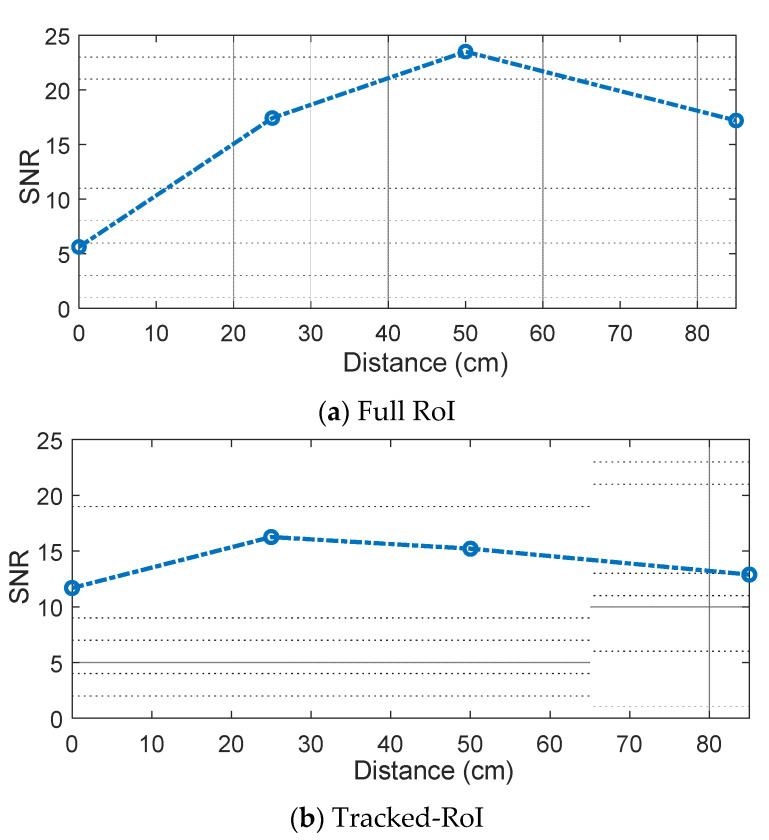
(**a**) SNR for full ROI using 2-PAM at the distance of 0 cm, 25 cm, 50 cm and 85 cm; (**b**) SNR for tracked ROI using 2-PAM at the distance of 0 cm, 25 cm, 50 cm and 85 cm.

Figure 10a,b shows the measured signal-to-noise ratio (SNR) of the full-RoI case and the tracked-RoI case at four different distances. The result of the full-RoI case shows that at the distance of 50 cm, the highest SNR of 23.5 dB is achieved. The SNR gradually decreases as the distance deviates from 50 cm. The SNR variation is in accordance with the light intensity captured by the camera, as shown in Figure 7. Figure 10b shows the SNR variation for the tracked RoI case. Overall, there is a smaller variation in the SNR distribution across the distances in this case. The distance of 25 cm shows the highest SNR of 16.3 dB. This is because, in the tracked-RoI case, the camera tracks and samples the brightest point with a faster frame rate.

**Figure 11:**
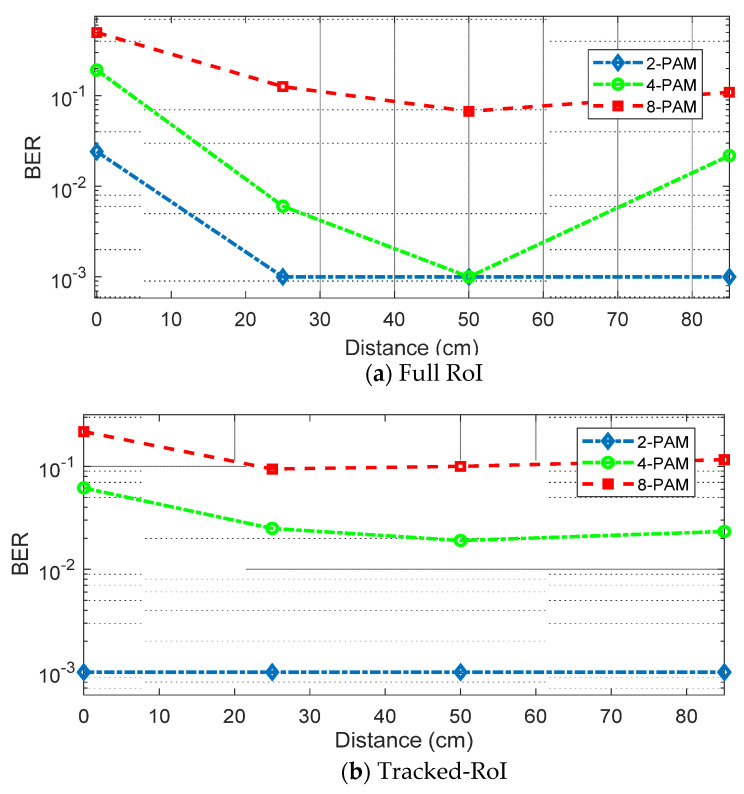
(**a**) BER for full RoI using 2-PAM, 4PAM and 8PAM without equalizer; (**b**) BER for tracked RoI using 2-PAM, 4-PAM and 8-PAM without equalizer.

Unlike the downlink test, it is difficult to change symbol rates under the limited frame rates of the optical camera. Hence, in this investigation, the depth of modulation level is changed. Figure 11a shows the BER performance at different distances in the case of full-RoI. 2-PAM, 4-PAM, and 8-PAM schemes are tested. The symbol rate of 17 Hz is applied. Actual data rates are 17 b/s, 34 b/s, and 51 b/s for 2-PAM, 4-PAM, and 8-PAM, respectively. Although 8-PAM can provide the highest data rate, the BER performance is poor due to insufficient SNR. In 4-PAM, reliable communication is possible at 50 cm where the SNR is the highest. For 2-PAM, BERs are less than 10^−3^ at all distances except for the case of 0 cm, as the SNR at this point is the worst due to the blockage.

**Figure 12:**
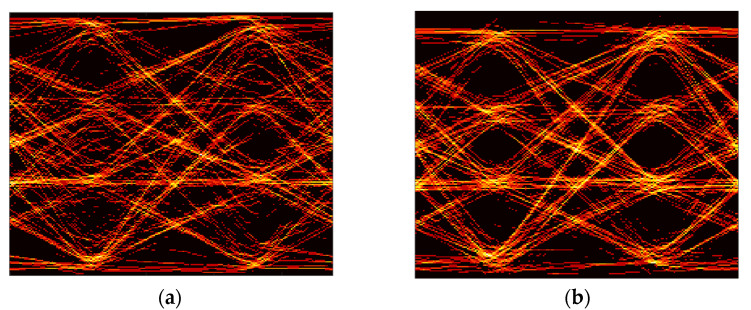
(**a**) Eye-diagram of the received 4-PAM signal of 840 b/s for the uplink communication at the distance of 25 cm without DFE for tracked ROI; (**b**) with DFE.

Figure 11b shows the BER for the tracked-RoI cases. The symbol rate of 420 Hz is applied. Actual data rates are 420 b/s, 840 b/s, and 1260 b/s for 2-PAM, 4-PAM, and 8-PAM, respectively. 2-PAM results show less than 10^−3^ at all distances (even at 0 cm). It means as long as there is a small portion of the reflected light to track and sample from the smart-farming unit, this method can reliably construct the uplink communication link. Next, to push the data rate further, the effectiveness of DFE on this optical camera-based receiver is tested. When the DFE is applied to 4-PAM results at 25 cm and 50 cm with relatively high SNRs in the case of tracked-RoI, a successful recovery with BER less than 10^−3^ is achieved. Figure 12a,b show the eye diagrams of the received 840 s b/s 4-PAM signal at 25 cm, before and after applying the DFE, showing a wider eye-opening in the equalized case.

## 4. Discussions and Conclusions

In this paper, we investigated the use of reflected light from the leafy vegetables after photosynthesis. A series of simulations and experimental results show the feasibility of this approach in constructing an energy-efficient RF-free environment. For the uplink, using the optical camera as a receiver, multiple smart-farming units can be monitored at the same time, exploiting the full-RoI. In addition, simultaneous reception of data from each smart-farming unit can be processed. Although there is a limited communication speed due to the innate low frame rate, adjusting RoI and tracking an individual smart-farming unit upon request enables a faster data rate with a higher frame rate. For the downlink, typical ceiling light with an APD was utilized. The maximum data rates achieved in the proof-of-concept demonstration are 840 b/s and 20 Mb/s for the uplink and downlink, respectively. Future work includes improving the data rate by applying more advanced modulation and multiplexing techniques and increasing the coverage by a larger FoV camera. Also, an interesting future topic to investigate is a large-scale demonstration to check the practicality of this work compared to the RF-smart-farming systems in terms of energy efficiency and growth and health of the vegetables.

## Figures and Tables

**Table 1:** Illuminance-PPFD conversion of tested RGB LEDs.

	Red LED	Green LED	Blue LED
Lux/PPFD	41.3	122.4	15.1

**Table 2:** Luminous efficacy of tested RGB LEDs.

	Red LED	Green LED	Blue LED
lm/W	217.3	543.9	58.7

**Table 3:** Required PPFD and illuminance and intensity for leafy vegetables [17,29].

Parameters	RGB Photon Flux (μmol·m^−2^ s^−1^)	Illuminance (lux)	Intensity (W/m^2^)
Total (400–700 nm)	250	14,208.6	52.1
Red (600–700 nm)	152.5	6298.3	29
Green (500–600 nm)	60	7344	13.5
Blue (400–500 nm)	37.5	566.3	9.6

**Table 4:** Geometrical and optical parameters for simulation.

Geometrical Parameters	Values
Room dimension	3 m × 2 m × 3 m
Center location	
Vegetableation unit (6 units) (length, width, height)	(0.4 m, 0.45 m, 0.8 m), (0.4 m, 1.55 m, 0.8 m) (1.5 m, 0.45 m, 0.8 m), (1.5 m, 1.55 m, 0.8 m) (2.6 m, 0.45 m, 0.8 m), (2.6 m, 1.55 m, 0.8 m)
Grow LED lights (6 units)	0.3 m above the vegetableation unit
Ceiling lights (6 units)	1.35 m above the vegetableation unit
Size	
Vegetableation area	0.3 × 0.3 m^2^
Grow LED lights	0.2 × 0.2 m^2^
Ceiling lights	0.2 × 0.2 m^2^
**Optical Parameters**	**Values**
Photodetector active area	1 cm^2^
Reflectance of vegetable (at green wavelength)	0.22 [30]
Lambertian order (ceiling and grow lights)	m = 1 (*Φ*_½_ = 60°)

## Data Availability

The data presented in this study are available on request from the corresponding author and on his personal website.

## References

[1] Kozai T. (2012). Sustainable plant factory: Closed plant production systems with artificial light for high resource use efficiencies and quality produce. Int. Symp. Soil. Cultiv..

[2] Mendez G.R., Mukhopadhyay S.C. (2013). A Wi-Fi based smart wireless sensor network for an agricultural environment. Wireless Sensor Networks and Ecological Monitoring.

[3] Kumar T.U., Periasamy A. (2021). IoT Based Smart Farming (E-FARM)’S. IJRAMR.

[4] Boonyopakom P., Thongna T. Environment Monitoring System through LoRaWAN for Smart Agriculture. Proceedings of the 5th International Conference on Information Technology (InCIT).

[5] Kalaivani T., Allirani A., Priya P. A survey on Zigbee based wireless sensor networks in agriculture. Proceedings of the 3rd International Conference on Trendz in Information Sciences & Computing (TISC2011).

[6] Harun A.N., Mohamed N., Ahmad R., Ani N.N. (2019). Improved Internet of Things (IoT) monitoring system for growth optimization of Brassica chinensis. Comput. Electron. Agric..

[7] Jagtap M., Shinde M.A., Tirodkar M.S., Patil M.A., Tambe M.A. (2017). Review Paper on Solar Powered & Arduino Controlled Agribot. IJSTE.

[8] Dai J., Sugano M. Low-Cost Sensor Network for Collecting Real-Time Data for Agriculture by Combining Energy Harvesting and LPWA Technology. Proceedings of the IEEE Global Humanitarian Technology Conference (GHTC).

[9] Havas M., Sheena Symington M. (2016). Effects of Wi-Fi radiation on germination and growth of broccoli, pea, red clover and garden cress seedlings: A partial replication study. Curr. Chem. Biol..

[10] Liptai P., Dolník B., Gumanová V. (2017). Effect of Wi-Fi radiation on seed germination and plant growth-experiment. Ann. Fac. Eng. Hunedoara.

[11] Kadam K., Chavan G.T., Chavan U., Shah R., Kumar P. (2016). Smart and Precision Polyhouse Farming Using Visible Light Communication and Internet of Things. Intelligent Computing and Information and Communication.

[12] Rehman S.U., Ullah S., Chong P.H.J., Yongchareon S., Komosny D. (2019). Visible Light Communication: A System Perspective—Overview and Challenges. Sensors.

[13] Singh D., Basu C., Meinhardt-Wollweber M., Roth B. (2015). LEDs for energy efficient greenhouse lighting. Renew. Sustain. Energy Rev..

[14] Lumoindong C.W.D., Muslim A., Nasreddin B.M., Galina M. (2018). Performance and environmental impacts review of Li-Fi and Wi-Fi technologies. J. Environ. Manag..

[15] Shetty A. (2016). A comparative study and analysis on Li-Fi and Wi-Fi. Int. J. Comput. Appl..

[16] Pennisi G., Orsini F., Blasioli S., Cellini A., Crepaldi A., Braschi I., Spinelli F., Nicola S., Fernandez J.A., Stanghellini C. (2019). Resource use efficiency of indoor lettuce (Lactuca sativa L.) cultivation as affected by red: Blue ratio provided by LED lighting. Sci. Rep..

[17] Kim H.H., Goins G.D., Wheeler R.M., Sager J.C. (2004). Green-light supplementation for enhanced lettuce growth under red-and blue-light-emitting diodes. HortScience.

[18] Lee S., Park S. Energy savings of home growing vegetables by using daylight and LED. Proceedings of the IEEE Sensors Applications Symposium Proceedings.

[19] Zukauskas A., Shur M.S., Gaska R. (2002). Introduction to Solid State Lighting.

[20] Ritchie R.J. (2010). Modelling photosynthetic photon flux density and maximum potential gross photosynthesis. Photosynthetica.

[21] Denault K.A., Cantore M., Nakamura S., DenBaars S.P., Seshadri R. (2013). Efficient and stable laser-driven white lighting. AIP Adv..

[22] Zhao T., Liu P., Zhang S., Ma Q. (2020). UAV assisted landing guided by UV LEDs. Appl. Opt..

[23] Chun H., Chiang C.J., O’Brien D.C. Visible light communication using OLEDs: Illumination and channel modeling. Proceedings of the 2012 International Workshop on Optical Wireless Communications (IWOW).

[24] Kahn J.M., Barry J.R. (1997). Wireless infrared communications. Proc. IEEE.

[25] Grubor J., Randel S., Langer K.D., Walewski J.W. (2008). Broadband information broadcasting using LED-based interior lighting. J. Light. Technol..

[26] Lighting Indoor Housevegetables|MU Extension. https://extension.missouri.edu/publications/g6515.

[27] Bian Z.H., Yang Q.C., Liu W.K. (2015). Effects of light quality on the accumulation of phytochemicals in vegetables produced in controlled environments: A review. J. Sci. Food Agric..

[28] Berkovich Y.A., Konovalova I.O., Smolyanina S.O., Erokhin A.N., Avercheva O.V., Bassarskaya E.M., Kochetova G.V., Zhigalova T.V., Yakovleva O.S., Tarakanov I.G. (2017). LED crop illumination inside space greenhouses. Reach.

[29] Pennisi G., Pistillo A., Orsini F., Cellini A., Spinelli F., Nicola S., Fernandez J.A., Crepaldi A., Gianquinto G., Marcelis L.F. (2020). Optimal light intensity for sustainable water and energy use in indoor cultivation of lettuce and basil under red and blue LEDs. Sci. Hortic..

[30] Altunbas S., Gozukara G., Sonmez N.K., Maltas A.S., Kaplan M. (2018). Relationship between spectral reflectance and vegetable nutrient element-chlorophyll content in lettuce (*Lactuca sativa* L.) growing. Fresenius Environ. Bull..

